# Transplantation of human villous trophoblasts preserves cardiac function in mice with acute myocardial infarction

**DOI:** 10.1111/jcmm.13165

**Published:** 2017-05-19

**Authors:** Zegen Wang, Ningzheng Dong, Yayan Niu, Zhiwei Zhang, Ce Zhang, Meng Liu, Tiantian Zhou, Qingyu Wu, Ke Cheng

**Affiliations:** ^1^ Cyrus Tang Hematology Center and Collaborative Innovation Center of Hematology Soochow University Suzhou China; ^2^ Thrombosis and Hemostasis Key Laboratory Ministry of Education Engineering Center for Hematological Disease Jiangsu Institute of Hematology the First Affiliated Hospital of Soochow University Suzhou China; ^3^ Molecular Cardiology Cleveland Clinic Cleveland OH USA; ^4^ Department of Molecular Biomedical Sciences and Comparative Medicine Institute North Carolina State University Raleigh NC USA; ^5^ Joint Department of Biomedical Engineering University of North Carolina at Chapel Hill and North Carolina State University Chapel Hill NC USA

**Keywords:** Human villous trophoblasts, myocardial infarction, cell therapy, paracrine effects

## Abstract

Over the past decade, cell therapies have provided promising strategies for the treatment of ischaemic cardiomyopathy. Particularly, the beneficial effects of stem cells, including bone marrow stem cells (BMSCs), endothelial progenitor cells (EPCs), mesenchymal stem cells (MSCs), embryonic stem cells (ESCs), and induced pluripotent stem cells (iPSCs), have been demonstrated by substantial preclinical and clinical studies. Nevertheless stem cell therapy is not always safe and effective. Hence, there is an urgent need for alternative sources of cells to promote cardiac regeneration. Human villous trophoblasts (HVTs) play key roles in embryonic implantation and placentation. In this study, we show that HVTs can promote tube formation of human umbilical vein endothelial cells (HUVECs) on Matrigel and enhance the resistance of neonatal rat cardiomyocytes (NRCMs) to oxidative stress *in vitro*. Delivery of HVTs to ischaemic area of heart preserved cardiac function and reduced fibrosis in a mouse model of acute myocardial infarction (AMI). Histological analysis revealed that transplantation of HVTs promoted angiogenesis in AMI mouse hearts. In addition, our data indicate that HVTs exert their therapeutic benefit through paracrine mechanisms. Meanwhile, injection of HVTs to mouse hearts did not elicit severe immune response. Taken together, our study demonstrates HVT may be used as a source for cell therapy or a tool to study cell‐derived soluble factors for AMI treatment.

## Introduction

AMI, also known as heart attack, leads to loss of cardiomyocytes, scar formation, ventricular remodelling and subsequent incurable heart failure 1, 2. Despite great advances in pharmacological and invasive treatment regimens, AMI causes high mortality and morbidity worldwide 3.

Cell‐based therapies offer a promising option for the treatment of AMI 4. And the beneficial effects of various cell types in AMI have been demonstrated by basic researches and clinical studies, which include skeletal myoblasts, BMSCs, EPCs, MSCs, ESCs, iPSCs, cardiac stem cells (CSCs) and cardiospheres 4, 5, 6, 7, 8, 9, 10, 11, 12, 13.

Stem cell therapy, however, is not always safe and effective 14. For example, transplantation of skeletal myoblasts in injured hearts can lead to severe arrhythmia which limits the further investigation of this cell type 15. Intramyocardial injection of pluripotent stem cells such as ESCs and iPSCs can lead to tumorigenicity and immunogenicity 16, 17. On the other hand, BMSC therapy is generally safe but recent meta‐analysis has revealed that the benefits of such therapy for AMI are only marginal 18. Intrinsic heart stem cells were identified one and half decades ago and such research has propelled the recent clinical trials using ckit‐positive CSCs or cardiosphere cells 11, 19. However, ckit‐positive cells are rare in the heart and the expansions of CSCs and cardiosphere cells are time‐consuming.

In consideration of all this, it is imperative to seek for new cell types to promote cardiac regeneration. Placental trophoblasts play pivotal roles at the maternal and foetal interface that is essential for nutrient and oxygen transfer 20. Trophoblasts can secrete a wide range of angiogenic factors and cytokines, such as vascular endothelial growth factor (VEGF) and insulin‐like growth factor (IGF) 21, 22. These factors have been reported to play essential roles in heart regeneration 23. Moreover, trophoblasts can produce Fas ligand to induces immune cell apoptosis 24. Furthermore, the expression of FSTL1 was also detected in primary term human villous 25. It is intriguing that the recombinant bacterial‐synthetized human Fstl1 protein promotes myocardial repair and regeneration by stimulating pre‐existing cardiomyocytes to re‐enter into cell cycle entry and division 26, 27, 28. In addition, human trophoblast injection did not cause tumour formation in nude mice 29.

Hence, we suggested that HVTs could secrete pro‐angiogenic and pro‐cardiogenic growth factors to promote heart regeneration after AMI. The data presented here provide new insights into the mechanisms of HVT regenerative biology and also form the foundation of future development of HVT‐based therapy for MI treatment.

## Materials and methods

### HVT culture and paracrine assay

HVTs (ScienCell Research Laboratories, San Diego, CA, USA) were maintained with trophoblast medium (ScienCell Research Laboratories) plus 5% FBS, 1% trophoblast growth supplement and 1% penicillin/streptomycin solution under 5% CO_2_ at 37°C, as described previously 30. When the cells reached 90% confluence, the culture medium was changed to serum‐free IMDM for 3 days for conditioning. Then, the conditioned medium (CM) from HVTs was harvested. Meanwhile, 5 × 10^5^ NRCMs isolated from 48‐hrs‐old Sprague Dawley rat pups 12 were plated onto fibronectin‐coated 12‐well plates (Corning, New York, USA) with 1 ml IMDM (Gibco, Waltham, MA, USA) containing 20% FBS (Hyclone, Logan, UT, USA) for 48 hrs. Then NRCMs were incubated with HVT‐derived CM, and plain IMDM as the control and injured with 100 μM H_2_O_2_. After 24 hrs, cells were fixed with 4% PFA and apoptotic cells were detected by terminal deoxynucleotidyl transferase dUTP nickend labelling (TUNEL) assay using the In Situ Cell Death Detection Kit (Roche Diagnostics, Indianapolis, IN, USA).

The pro‐angiogenic effects of HVT‐CM were studied in an endothelial cell tube formation assay. HUVECs; from ATCC were seeded onto growth factor‐reduced Matrigel™ (BD Biosciences, New York, USA) in 96‐well plates at a density of 2 × 10^4^ cells per well. 100 μl HVT‐CM or DMEM (as control) was added into the wells. Within 6 hrs, the wells were imaged with a Leica (Wetzlar, Germany) white light microscope. The average tube length was then measured with NIH Image J Software (NIH, Bethesda, MD, USA).

The presence of various proteins in the CM was assayed with a human angiogenesis antibody array (RayBiotech, Norcross, GA, USA).

### RT‐PCR

Total RNA was extracted from HVT cell pellets using a High Pure RNA Isolation Kit (OMEGA bio‐tek, Norcross, GA, USA), according to the manufacturer's instructions. The first strand of cDNA synthesis from RNA template was performed with cDNA reverse transcription kit (Thermo, Logan, UT, USA), according to manufacturer's instructions. The sequences of the primers used in this study were listed as follows: for α‐hCG, forward primer AACCCATTCTTCTCCCAGCC, reverse primer GCCGTGTGGTTCTCCACTTT; for β‐hCG, forward primer ATGTGCGCTTCGAGTCCATC, reverse primer GGGCCTTTGAGGAAGAGGAG; for angiogenin, forward primer CCTGACCTCACCCTGCAAAG, reverse primer GCTCGGTACTGGCATGGAG; for CXCR4, forward primer TCATCACGCTTCCCTTCTGG, reverse primer CCACCTTTTCAGCCAACAGC.

### Animal procedures

All animal experiments were performed in accordance with the experimental protocol approved by Soochow University Laboratory Animal Center. The AMI model was performed as described before 31. Briefly, male C57BL6 mice were anaesthetized with 3% isoflurane inhalation. A small skin cut and a purse suture were made over the left thoracic wall. After that, a small incision was made at the fourth intercostal space. The heart was then gently popped out through the window. The left main descending coronary artery (LCA) was ligated at a site 3 mm from its origin. After the ligation, one million of HVTs were intramyocardially injected into the border zone of the mice immediately. Then the heart was placed back into the intrathoracic space with fast air evacuation and chest wall closure, by the previously placed purse‐string suture. All animals received buprenorphine for analgesia after the surgery.

### Echocardiography

Mice were anaesthetized with a 1.5% isoflurane–oxygen mixture at baseline (4 hrs post‐MI) and 4 weeks afterwards. And two‐dimensional short axis images were recorded using Vevo2100 System (Visual Sonics, Toronto, ON, Canada).

### Tissue harvesting

Mice were killed at various time‐points (24 hrs for TUNEL staining, 7 days for CD8 staining and 28 days for histology) after treatment, and then the hearts were harvested and snap‐frozen in optimal cutting temperature (OCT) compound (Leica) at −30°C for cryosectioning and histology or immersed to liquid nitrogen for Western blotting.

### Western blotting

Heart tissues stored in liquid nitrogen were lysed using RIPA buffer (Beyotime, Nantong, Jiangsu, China). Protein concentrations were measured by the BCA method (Thermo). To separate proteins, equal amounts of protein samples were loaded to SDS‐PAGE gels, separated and then transferred onto PVDF membranes (Millipore, Billerica, MA, USA). Then the membranes were incubated with primary antibodies against VEGF, hepatocyte growth factor (HGF), insulin‐like growth factor‐1 (IGF1) and GAPDH, respectively. This was followed by blotting with HRP‐conjugated secondary antibodies. Immunoreactive bands were visualized by enhanced chemiluminescence on exposed X‐ray films.

### Masson's trichrome staining

Masson's trichrome stain was performed as described 32. In brief, sections were immersed in Bouin's solution overnight. Slides were then rinsed for 10 min. under running water to remove excessive picric acid. Then the slides were transferred to Weigert's haematoxylin solution for 5 min. Slides were then rinsed and stained with scarlet‐acid fuchsin for 5 min. and rinsed again. Slides were then stained with phosphotungstic/phosphomolybdic for 10 min. and transferred directly into aniline blue solution for 5 min., and 2% acetic acid for 2 min. each. Slides were then rinsed, dried, and mounted using DPX mounting media.

### Histology

Hearts were sectioned at a thickness of 3 μm for immunofluorescence staining. The sections were first blocked with 5% BSA in PBS and then stained with anti‐von Willebrand factor (Abcam Cambridge, MA, USA), anti‐human nuclei antigen (Millipore) and CD8 (Bio‐Rad, Hercules, CA, USA) antibodies. DyLight 488‐ or 594‐conjugated secondary antibodies (Immunoreagents, NC, Raleigh, NC, USA) were used in conjunction with these primary antibodies. Images were taken by a Zeiss (Olympus, Tokyo, Japan) confocal microscopy system. Apoptotic cells in heart tissues were detected by In Situ Cell Apoptosis Detection Kit, FITC (Sangon, Shanghai, China).

### Statistical analysis

Results are presented as mean ± standard deviation (S.D.). Comparisons between PBS and HVT groups were performed with two‐tailed unpaired Student's *t*‐test. Differences were considered statistically significant when *P* < 0.05.

## Results

### Characterization of human HVTs

**Figure 1:**
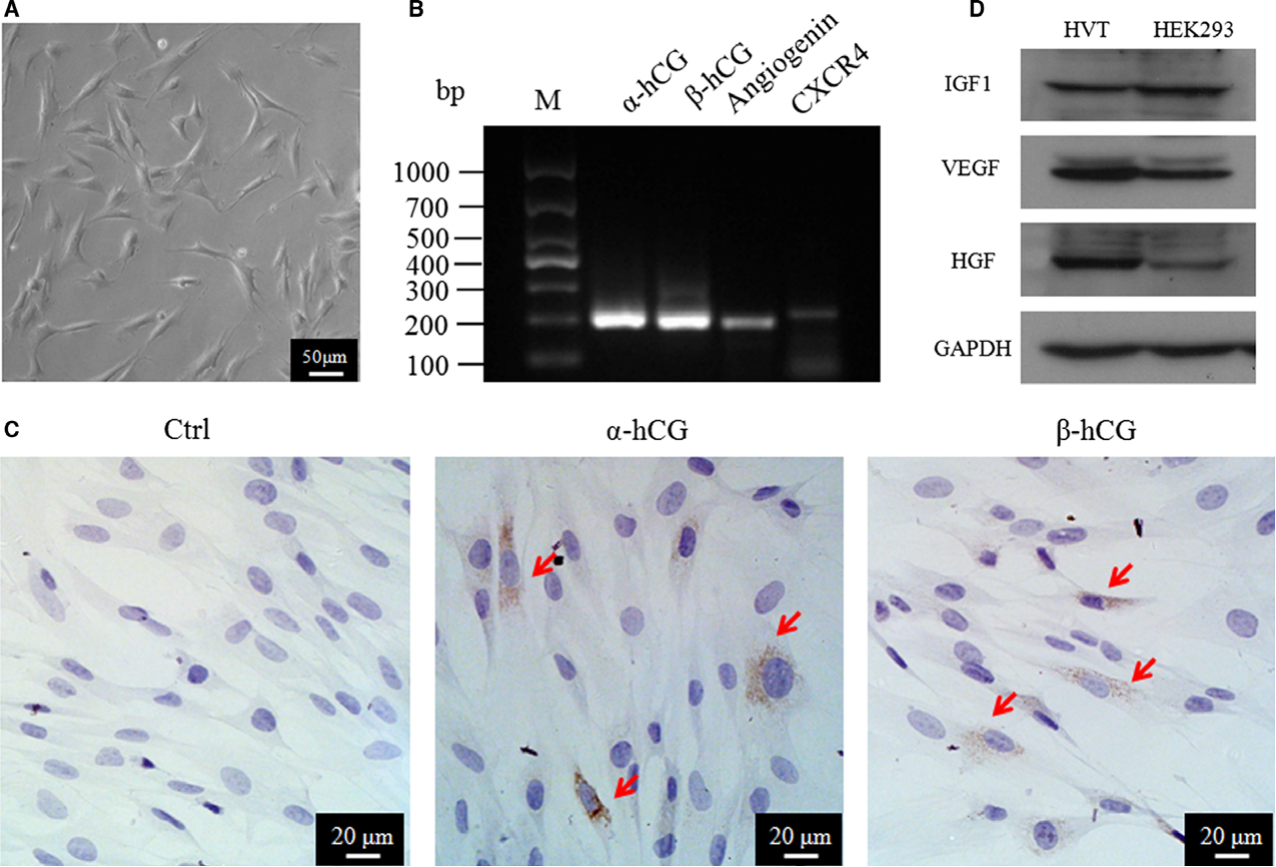
Characterization of HVTs. (**A**) Morphology of HVTs in trophoblast medium (TM) after 24 hrs. (**B**) Transcription of RNAs by RT‐PCR in HVTs. (**C**,** D**) Protein expression of HVTs by immunofluorescence or Western blotting. HEK293 cell lysate is a positive control in D.

To confirm the identity of human HVTs, the RNA and protein expressions of widely accepted HVT markers were examined. As indicated in Figure 1A, HVT cells exhibited a typical multiangular morphology as descriptions by Graham *et al*. 33 under our culture conditions. Expressions of α/β‐hCG, CXCR4 and angiogenin were detected by RT‐PCR (Fig. 1B). Meanwhile, immunocytochemical staining confirmed the α/β‐hCG expression in HVTs (Fig. 1C). Moreover, VEGF, HGF and IGF‐1 proteins were detected in HVT cell lysates (Fig. 1D) by Western blotting. These compound data sets confirmed the identity of HVTs.

### Paracrine assays on HVTs

**Figure 2:**
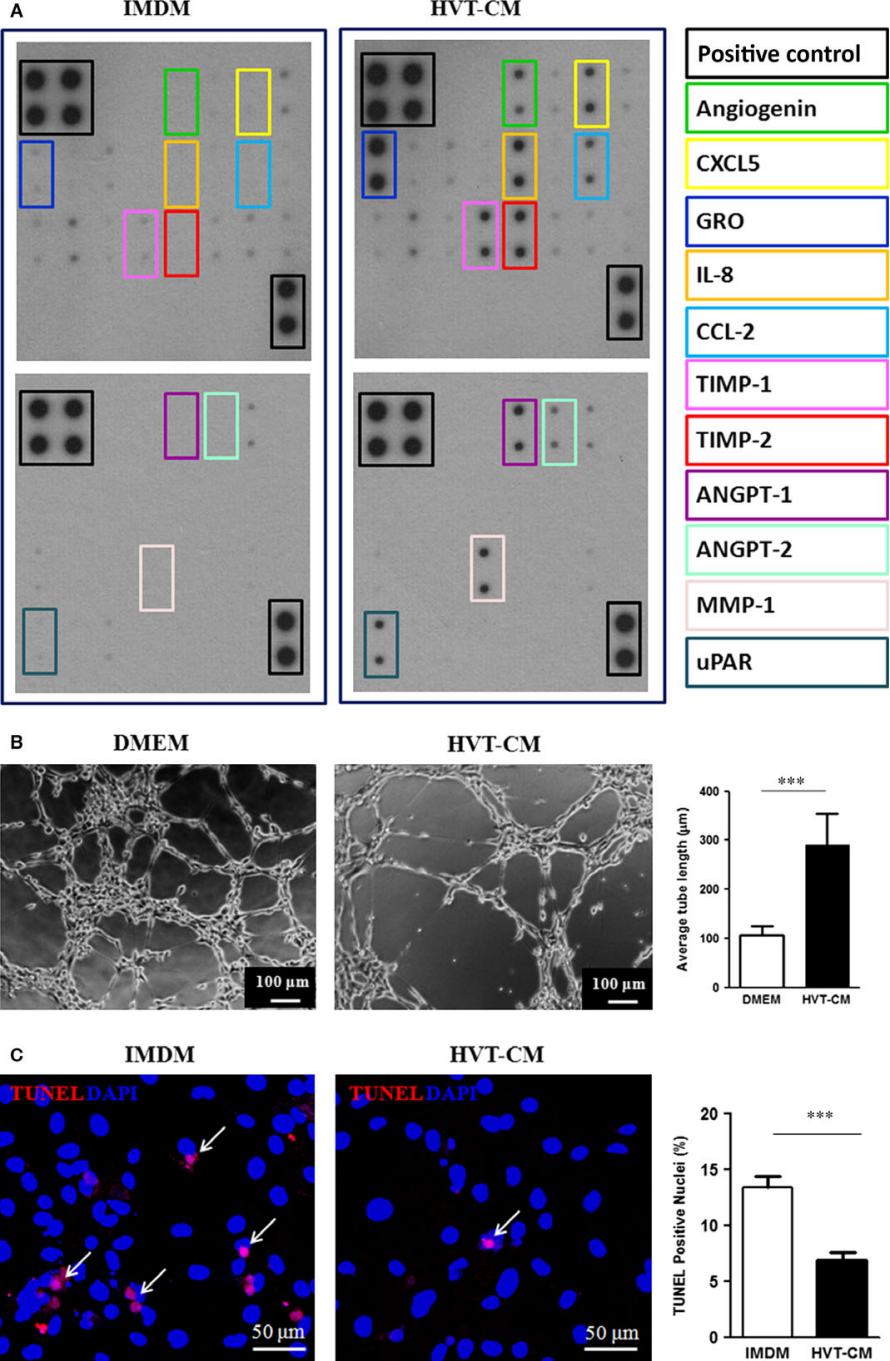
Paracrine assays, HUVEC tube formation and NRCM apoptosis in HVT‐CM. (**A**) Representative antibody array images showing the proteins present in HVT‐CM and control TM. Abbreviations: CM, conditioned media; TM: trophoblast medium; CXCL5, C‐X‐C motif chemokine 5; GRO: growth‐regulated protein; IL8: interleukin‐8; CCL2: C‐C motif chemokine 2; TIMP‐1: Metalloproteinase inhibitor 1; TIMP‐2: Metalloproteinase inhibitor 2; ANGPT‐1: Angiopoietin‐1; ANFPT‐: Angiopoietin‐2; MMP‐1: Interstitial collagenase; uPAR: Urokinase plasminogen activator surface receptor. (**B**) Representative tube formation by HUVECs on Matrigel incubated with HVT‐CM or control DMEM (*n* = 12–14). (**C**) Representative confocal images showing NRCMs exposed to 100 μM H_2_O_2_ for 24 hrs in HVT‐CM or control IMDM. Apoptotic cells were detected by TUNEL staining and quantified (*n* = 5). ***indicates P<0.001 when HVT group compared to PBS. Data are presented as mean ± S.D.

A cytokine array (Fig. 2A) revealed that HVTs secreted pro‐angiogenic factors such as angiogenin, angiopoietin‐1 (ANGPT1), angiopoietin‐2 (ANGPT2) and metalloproteinase inhibitor‐1/2 (TIMP‐1/2). Given the presence of the pro‐angiogenic growth factors in HVT cell lysates (Fig. 1D) and the result of paracrine assays of HVT‐derived CM, we showed that CM from HVTs could promote tube formation of endothelial cells *in vitro*. When incubated with HVT‐CM, the tube formation of HUVECs on Matrigel™ was enhanced within 6 hrs (Fig. 2B), as compared to the tubes formed in control DMEM. Furthermore, HVT‐CM enhanced the resistance of NRCMs to oxidative stress. After exposure to 100 μM H_2_O_2_ for 24 hrs, the number of TUNEL‐positive cells was lower in the HVT‐CM group than that in the control IMDM group (Fig. 2C).

### Intramyocardial injection of HVTs improves cardiac function and decreases infarct size

**Figure 3:**
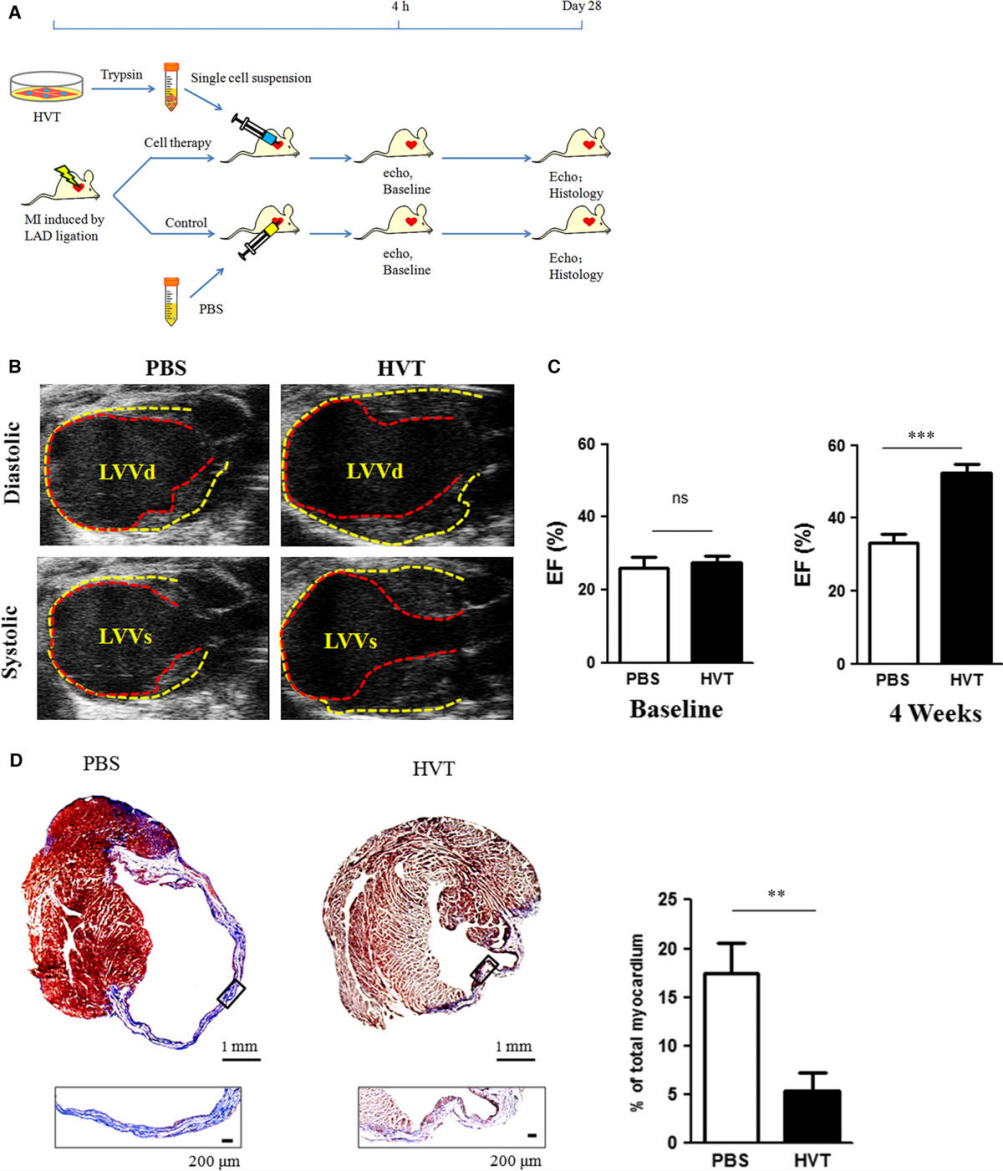
Cardiac function and fibrosis. (**A**) Schematic diagram showing the animal procedures. (**B**) Representative echocardiography images at 4 weeks after treatment. The pericardium and endocardium are outlined with yellow and red dotted lines, respectively. LVVd, left ventricular volume in diastole; LVVs, left ventricular volume in systole. (**C**) Left ventricular ejection fraction (LVEF) measured by echocardiography at baseline (left) and 4 weeks afterwards (right) in PBS or HVT groups (*n* = 7–9 mice per group). Baseline LVEFs were indistinguishable between the two groups. (**D**) Representative Masson's trichrome‐stained images and quantification of fibrotic area of the infarcted myocardium 4 weeks after treatments (*n* = 4 mice per group). Scar tissue and viable myocardium are identified by the blue and red colours, respectively. Snapshots of the infarct border zone (black box area) are presented beneath each group. **indicates P<0.01 when HVT group compared to PBS., ***indicates P<0.001 when HVT group compared to PBS. Data are presented as mean ± S.D.

The animal study design is outlined in Figure 3A. To determine the cardiac function, echocardiography was performed at 4 hrs (baseline) and 28 days post‐MI (Fig. 3A). Heart morphometry at 4 weeks showed HVT injection reduced left ventricular (LV) chamber dilation as compared to PBS‐treated mice (Fig. 3B). The data indicated that HVT treatment protected cardiac functions while the cardiac functions in PBS‐treated mice continued to deteriorate (Fig. 3C). We reasoned that part of HVT therapeutic effects on cardiac function may come from decreased scar size. Thus, fibrosis was analysed by Masson's trichrome staining. Compared to PBS‐injected hearts, HVT treatment significantly inhibited scar formation (Fig. 3D). These data conferred the therapeutic benefits of HVTs in our mouse models of AMI.

### HVTs attenuate tissue apoptosis and promote angiogenesis

**Figure 4:**
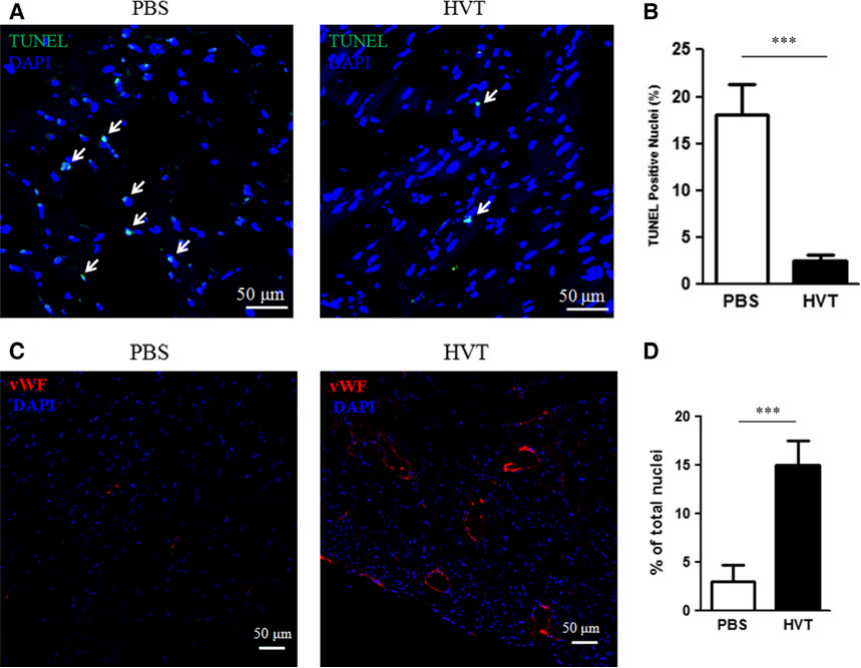
Reduced apoptosis and increased angiogenesis. (**A**) Microscopy images of TUNEL staining in heart sections of HVT‐ or PBS‐treated mice 8 hrs after MI. Apoptotic cells (red colour) are highlighted with white arrowheads. (**B**) Quantitation of apoptotic cells (*n* = 4). (**C**) Representative micrographs showing vWF‐stained vasculatures in the PBS and HVT groups 4 weeks after treatment. (**D**) Quantification of vWF‐stained vasculatures (*n* = 3 mice per group). ***indicates P<0.001 when HVT group compared to PBS. Data are presented as mean ± S.D.

To test the cardioprotective effects of HVTs, we performed TUNEL staining to evaluate myocardial apoptosis 24 hrs after the treatment. HVT injection reduced TUNEL‐positive nuclei (Figs. 4A and B). These data sets manifest a cardioprotective effect of HVT treatment post‐MI.

As CM from HVTs promoted the tube formation of HUVECs *in vitro* (Fig. 2A), we showed that transplantation of HVTs can stimulate neovascularization in post‐MI hearts. vWF‐positive capillary density was strikingly higher in the HVT‐treated group than that in the PBS control‐treated group (Figs. 4C and D).

### HVTs do not graft permanently or trigger immune reactions in the mouse heart

**Figure 5:**
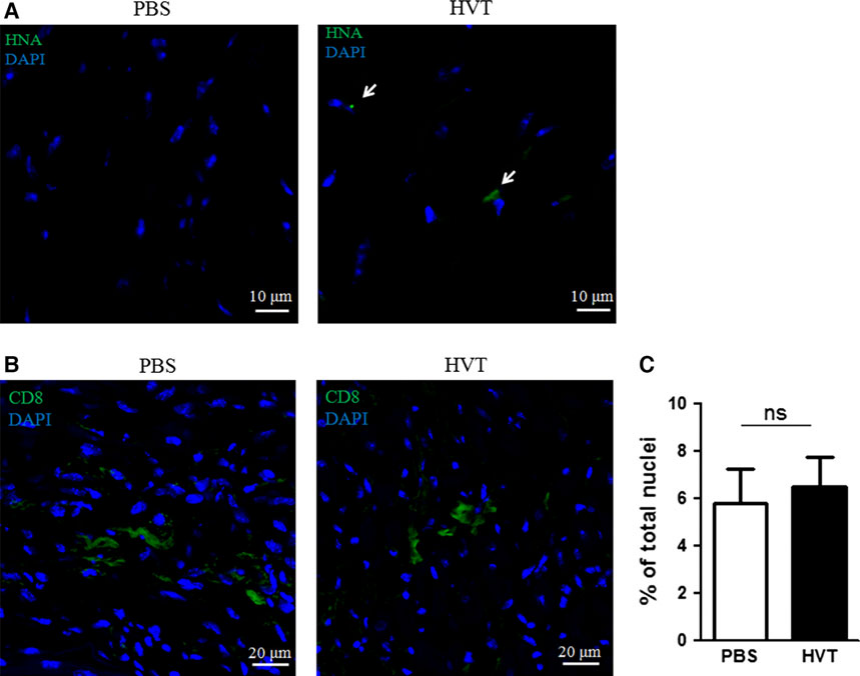
Cell retention and immune response in heart tissues. (**A**) Representative confocal images showing the cell retention of HVTs (positive for human nuclei antigen (HNA); green, indicated by white arrows) in the infarct border zone. (**B**) Representative confocal images showing CD8‐positive cells in the infarct border zone. (**C**) Quantification of CD8‐positive cells in the infarct border zone. ns indicated there was no significant difference when HVT group compared to PBS. Data are presented as mean ± S.D.

Although a few HNA^+^ cells could be detected in the cell‐injected hearts (Fig. 5A), the small numbers of engrafted cells seemed not sufficient to support the observed therapeutic benefits. This suggested that HVTs exerted cardiac protection through paracrine effects rather than direct engraftment and differentiation 34.

To evaluate the immunogenicity of such xenogenic transplantation of HVTs 35, immunofluorescent staining of immune cells was performed 7 days after cell injection. The numbers of CD8‐positive T cells were indistinguishable between the HVT and PBS treatment groups (Fig. 5B and C), suggesting that injection of human HVTs did not trigger T‐cell infiltration in the mouse heart.

## Discussion

Cardiovascular disease (CVD) especially MI is the major cause of morbidity and mortality worldwide 3. The ischaemia often results in irreversible cardiomyocyte death, which further translates into inflammation, fibrosis and deterioration of cardiac function, and ultimately leads to heart failure 36. Despite tremendous advances in modern medical therapy, the prognosis for heart failure patients remains unsatisfactory. Every year there are still a substantial number of patients suffering from progressive heart failure as a result of myocardium injury.

The last decade witnessed a burst of cell therapy trials for heart diseases. Various sources of cells were studied to treat MI. These cells include telocytes, skeletal myoblasts, BMSCs, EPCs, MSCs, ESCs and iPSCs 1, 37, 38. Intrinsic heart stem cells in MI treatment research have propelled the recent clinical trials using ckit‐positive CSCs or cardiosphere‐derived cells 11, 19.

Nevertheless, there is an urgent need for alternative cells sources to promote cardiac regeneration. HVTs can be readily isolated from human placental villous. Comparing to stem cells, HVTs can be readily grown into a large quantity, avoiding ethical and logistical issues as harvesting of HVTs does not harm the mother or the infant. Overall, the HVTs encompass several properties, as described above, which encourage their utilization in the cell transplantation for cardiac repair.

Our present results demonstrated that HVTs are capable of promoting angiogenesis, reducing apoptosis and fibrosis, and improving cardiac function in mice with AMI.

We found that CM collected from HVTs can promote the tube formation of HUVECs on Matrigel™ and enhance the resistance of rat cardiomyocytes to oxidative stress *in vitro*. After injection, only a few of HVTs engrafted in the mouse heart. This might indicate that injected cells contributed to therapeutic benefits mainly through paracrine effects, for example secretion of regenerative factors. It has been reported that trophoblasts produce various factors including VEGF, SDF‐1, HGF and IGF‐1 39. These factors have been shown to play essential roles in heart regeneration. VEGF is a pro‐angiogenic agent 40. SDF‐1 augments EPCs recruitment and haematopoietic stem cells (HSCs) mobilization and homing to ischaemic sites, subsequent promoting neovascularization in infarct area 41, 42. HGF has mitogenic, morphogenic, and anti‐apoptotic effects and plays pivotal roles in regeneration and protection of organs such as liver, kidney and lung and reduces ischaemia/reperfusion injury in AMI hearts directly by suppressing cardiomyocyte cell death 43, and IGF‐1 is a potent pro‐survival factor, which has been shown to reduce myocyte apoptosis and ventricular dilation after infarction 44. In addition, it has been reported that genes related to apoptosis inhibition were up‐regulated in first trimester placental villi 45, which may contribute to cardiomyocyte survival after ischaemia.

Xenogenic/allogeneic cell transplantation can lead to potential graft‐versus‐host disease 35. Here we showed that there were no significant differences in the numbers of CD8‐positive T cells between the HVT and PBS treatment groups in immunocompetent (normal) mice. This is consistent with the previous observations regarding the immunomodulatory effects of HVTs. During pregnancy, to prevent deleterious immune response towards the conceptus, trophoblasts induce tolerance of the foetal allograft against maternal immune system through Fas–FasL interactions 46, 47.

## Conclusion

Taken together, this study demonstrates the regenerative potential of HVTs in AMI. Such therapeutic benefits are mediated by the pro‐angiogenic, anti‐apoptotic and anti‐fibrotic effects of HVTs. Therefore, HVTs may be used an alternative cell source for the treatment of MI.

## Conflicts of interest

The authors confirm that there are no conflicts of interest.
